# An advanced, highly sensitive and versatile *Cypridina* luciferase-based HPV pseudovirus system for broad-spectrum neutralization and non-invasive *In vivo* monitoring

**DOI:** 10.3389/fimmu.2026.1863344

**Published:** 2026-05-29

**Authors:** Hyemi Kim, Chai Won Kim, Kihyuck Kwak

**Affiliations:** 1 Department of Microbiology and Immunology, Yonsei University College of Medicine, Seoul, Republic of Korea; 2 Brain Korea 21 Project for Medical Science, Yonsei University College of Medicine, Seoul, Republic of Korea; 3 Institute for Immunology and Immunological Diseases, Yonsei University College of Medicine, Seoul, Republic of Korea

**Keywords:** *Cypridina* luciferase, high-throughput screening, human papillomavirus (HPV), *in vivo* imaging, multivalent vaccine, neutralization assay, pseudovirus

## Abstract

**Introduction:**

Evaluating next-generation prophylactic human papillomavirus (HPV) vaccines requires high-throughput and scalable neutralization assays spanning a wide array of genotypes. Standard secreted alkaline phosphatase (SEAP)-based platforms, however, are limited by tedious protocols, such as mandatory serum heat inactivation, and restricted sensitivity. To address these bottlenecks, we developed an advanced pseudovirus (PsV) platform using Cypridina luciferase (cLuc), a versatile dual-reporter system that facilitates both extracellular secretion and intracellular monitoring.

**Methods:**

A comprehensive library of cLuc-engineered PsVs was constructed, covering 28 distinct mucosal and cutaneous HPV genotypes. We verified their structural integrity and antigenic presentation via neutralization profiling with L2 N-terminus-specific monoclonal antibodies (JWW-1/JWW-2).

**Results:**

The cLuc-PsVs demonstrated robust infectivity along with excellent analytical linearity. Critically, this system eliminated the need for thermal inactivation of sera while maintaining an exceptional correlation (R² > 0.99) with traditional SEAP assays during evaluations of hyperimmune mouse sera. Beyond in vitro screens, the platform's translational value was confirmed in a murine cervicovaginal challenge model, where real-time bioluminescence imaging enabled non-invasive tracking of mucosal infection across all 28 genotypes.

**Discussion:**

This dual-purpose cLuc-PsV platform provides a highly efficient, sensitive alternative to conventional neutralization assays. By unifying high-throughput in vitro quantification with longitudinal in vivo imaging, this system offers a robust tool to accelerate the preclinical screening of multivalent HPV vaccine candidates.

## 1 Introduction

Human papillomavirus (HPV) represents a formidable and pervasive global health threat, acting as the primary etiologic agent for virtually all cervical carcinomas—a leading cause of cancer-associated mortality in women worldwide—as well as driving a substantial and growing subset of other anogenital and oropharyngeal malignancies (1). The extensive global deployment of prophylactic HPV vaccines, which are formulated from recombinantly expressed major capsid protein L1 that self-assembles into highly immunogenic virus-like particles (VLPs), has profoundly decreased the prevalence of targeted HPV infections and their corresponding precancerous lesions. Because vaccine-induced immunity and long-term protection are predominantly mediated by type-specific neutralizing antibodies (2), the precise, scalable, and high-throughput quantification of these humoral immune responses is of paramount importance. As the field advances from bivalent and quadrivalent formulations to nonavalent and potentially broader multivalent vaccines, robust analytical systems are urgently required (3). These systems are essential not only to define the immunological correlates of protection for next-generation vaccines, but also to conduct large-scale epidemiological surveillance to monitor herd immunity, dose-sparing schedules (e.g., single-dose regimens), and potential genotype replacement dynamics in vaccinated populations.

At present, the *in vitro* pseudovirus-based neutralization assay (PBNA) remains the universally accepted gold standard for quantifying HPV-specific neutralizing antibodies (4, 5). This well-established assay employs engineered pseudoviruses (PsVs) comprising the L1 major and L2 minor capsid proteins that encapsidate a detectable reporter gene. By closely mimicking the initial receptor-binding to heparan sulfate proteoglycans (HSPGs), subsequent conformational changes, and L2-mediated endosomal escape mechanisms characteristic of natural HPV infections, the PBNA provides a highly biologically relevant readout (6, 7). Historically, secreted alkaline phosphatase (SEAP) or conventional intracellular luciferases (such as Firefly and Renilla) have served as the primary reporters for these platforms (8). Nevertheless, despite their widespread use over the past two decades, these legacy systems are hampered by notable methodological flaws that restrict their modern utility.

Although SEAP assays benefit from an extracellular readout that preserves cell viability, they suffer from a restricted dynamic range and mandate a rigorous 65°C thermal inactivation step. This heating process is critical to suppress endogenous alkaline phosphatase activity inherent in mammalian cells and serum samples. However, incomplete inactivation frequently leads to persistent background interference, which degrades signal clarity at high serum dilutions. Coupled with a prolonged colorimetric development phase, this procedural burden significantly restricts the assay’s scalability for massive clinical cohort evaluations and introduces thermal-induced experimental artifacts. Conversely, traditional intracellular luciferase formats necessitate tedious, destructive cell lysis protocols prior to signal detection. This invasive step entirely eliminates the possibility of longitudinal, real-time kinetic tracking, inherently increases inter-well variability due to inconsistent lysis efficiency, and obstructs automated high-throughput workflows.

To decisively overcome these persistent technical obstacles, *Cypridina* luciferase (cLuc), a highly efficient enzyme isolated from the marine ostracod *Cypridina hilgendorfii*, has emerged as a strategically superior alternative. Functioning independently of ATP, this naturally secreted reporter exhibits a highly advantageous dual-distribution profile: roughly 70–80% of the synthesized enzyme is actively released into the extracellular environment via the classical secretory pathway, while the remaining 20–30% is transiently retained within the host cell (9). Upon interaction with its specific imidazopyrazinone substrate, vargulin, cLuc generates an intensely bright, flash-type luminescent signal. For *in vitro* serological applications, this unique biochemical signature allows for the immediate, highly sensitive quantification of viral infectivity directly from minute aliquots of culture supernatants, entirely bypassing the destructive cell lysis process. Consequently, it fully preserves cell viability, completely eliminates the problematic and variable thermal inactivation step required in SEAP assays, and dramatically streamlines experimental workflows. Moreover, the superior signal intensity of cLuc confers exceptional analytical sensitivity and a remarkably broad linear dynamic range, facilitating the precise detection of even trace levels of viral infection without optical saturation (10).

Crucially, the dual-localization of cLuc provides unparalleled methodological versatility. While the robust secreted fraction optimizes automated, high-throughput *in vitro* neutralization assays, the intracellularly retained portion enables precise, non-invasive spatiotemporal monitoring during *in vivo* mucosal challenge studies using advanced bioluminescence imaging. The primary objective of the present investigation was to design, optimize, and rigorously validate this highly sensitive cLuc-based PBNA platform across a broad phylogenetic spectrum of HPV. To this end, we successfully constructed a comprehensive panel of cLuc-PsVs representing 28 distinct high-risk and low-risk HPV genotypes. We further verified the structural authenticity and stoichiometric accuracy of this diverse library by confirming the functional incorporation of the L2 minor capsid protein via cross-reactivity with JWW-1 and JWW-2, monoclonal antibodies targeting a highly conserved neutralizing epitope (11). Our compelling findings confirm that the cLuc-based platform not only delivers superior operational reproducibility and exceptional analytical concordance with the traditional SEAP assay *in vitro*, but also flawlessly translates into a Depo-Provera-sensitized murine cervicovaginal challenge model, enabling quantitative, real-time *in vivo* tracking across all 28 genotypes.

## 2 Materials and methods

### 2.1 Cell culture and animals

HEK293TT cells, specifically engineered to overexpress the SV40 large T antigen to facilitate high-yield episomal plasmid replication, were cultured in Dulbecco’s Modified Eagle Medium (DMEM; HyClone, Logan, UT, USA) supplemented with 10% fetal bovine serum (FBS; HyClone) and 1% penicillin-streptomycin. Cultures were maintained in a humidified 37°C incubator with 5% CO_2_. For the scalable production of VLPs, Expi293F cells (Thermo Fisher Scientific, Waltham, MA, USA) were grown in suspension using serum-free, chemically defined Expi293 Expression Medium under 8% CO_2_ at 37 °C on an orbital shaker rotating at 125 rpm, optimizing gas exchange and cell density. Six-week-old female BALB/c mice (Orient Bio, Seongnam, Republic of Korea) were housed under tightly regulated specific pathogen-free (SPF) conditions and acclimatized for seven days prior to experimental procedures. All *in vivo* protocols strictly complied with ethical guidelines and were formally approved by the Institutional Animal Care and Use Committee (IACUC) of Yonsei University College of Medicine (Approval No. IACUC-2021-0188).

### 2.2 Preparation of plasmids

The pCMV-*cypridina* reporter plasmid, driven by a robust cytomegalovirus immediate-early promoter for maximal gene expression, was acquired from Thermo Fisher Scientific. Plasmids encoding the conventional SEAP reporter, as well as the codon-optimized L1 and L2 capsid proteins encompassing the diverse 28 HPV genotypes, were sourced from Addgene (Watertown, MA, USA).

### 2.3 Production of HPV PsVs and VLPs

To synthesize reporter-encapsidating PsVs, HEK293TT cells were efficiently co-transfected with HPV L1 and L2 expression plasmids alongside either the pCMV-*cypridina* or SEAP reporter construct using the TurboFect transfection reagent (Thermo Fisher Scientific). Following a 48-hour incubation period to allow for sufficient protein expression and preliminary assembly, cells were harvested, washed with MgCl_2_-supplemented DPBS (DPBS-Mg), and lysed in a specialized buffer containing 0.5% (w/v) Brij58 and 0.2% (v/v) RNase cocktail. The non-ionic detergent Brij58 gently permeabilizes the cellular membrane without denaturing the assembling capsids, while the RNase eliminates competing cellular RNA, ensuring preferential packaging of the reporter plasmid. Cell lysates were subsequently incubated at 37°C for 24 hours to facilitate the essential disulfide bond formations requisite for *in vitro* capsid maturation (12). Mature PsVs were clarified by low-speed centrifugation (10,000× g, 10min) to remove bulk cellular debris and subsequently purified through a three-tier OptiPrep density gradient (27%, 33%, and 39% w/v). Ultracentrifugation at 280,000× g for 16 hours at 16 C using an SW41 Ti rotor (Beckman Coulter) precisely separated fully matured, reporter-packaged virions from empty capsids and residual host proteins. Fractions demonstrating peak infectivity were retained. Vaccine-grade VLPs were generated similarly in Expi293F cells utilizing the ExpiFectamine 293 reagent (Thermo Fisher Scientific), intentionally omitting the reporter plasmids to produce empty, highly immunogenic antigen particles. All purified viral constructs were meticulously aliquoted and cryopreserved at −80°C to preserve structural integrity.

### 2.4 Production of JWW-1 and JWW-2 monoclonal antibodies

The broadly cross-reactive human chimeric monoclonal antibodies JWW-1 and JWW-2 were produced by transiently co-transfecting Expi293F suspension cells with their respective heavy and light chain expression plasmids. Following a 7-day incubation, the secreted antibodies were harvested from the clarified supernatant, highly purified via Protein A/G affinity chromatography (Thermo Fisher Scientific), and concentrated using Amicon Ultra centrifugal filters (Millipore, Burlington, MA, USA) prior to precise spectrophotometric quantification.

### 2.5 SDS-PAGE and western blot analysis

To thoroughly assess viral capsid integrity and correct stoichiometric assembly, purified PsV fractions were strongly denatured in 4× SDS sample buffer containing 2% β-mercaptoethanol at 95 C for 5 minutes. The denatured proteins were separated on 10% polyacrylamide gels and visualized using InstantBlue^®^ Coomassie Protein Stain (Abcam, Cambridge, UK) to objectively confirm the proportional integration of L1 (~55 kDa) and L2 (~72 kDa) proteins. For highly sensitive immunoblotting, resolved proteins were transferred to polyvinylidene fluoride (PVDF) membranes (Millipore). The membranes were blocked with 5% non-fat dried milk in Tris-buffered saline containing 0.1% Tween-20 (TBST) for 1 hour to prevent non-specific binding, and probed overnight at 4°C with the primary antibodies JWW-1 or JWW-2 (1:1000 dilution). Following exhaustive TBST washes, membranes were incubated with an HRP-conjugated Goat anti-Human IgG Fc secondary antibody (Invitrogen) for 1 hour. Immunoreactive bands were visualized using the enhanced WesternBright ECL Kit (Advansta Inc.) and digitally captured with a Solo6X Edge imaging system (Vilber Lourmat).

### 2.6 Mouse immunization and serum collection

To generate robust hyperimmune sera mimicking a strong vaccine response, female BALB/c mice received intramuscular (i.m.) injections into the right hind limb. Each 100 µL dose comprised 2 µg of multivalent VLPs optimally formulated with Alhydrogel adjuvant (InvivoGen, San Diego, CA, USA) to stimulate a Th2-skewed humoral immune response (13). The intensive immunization schedule consisted of three doses administered at strictly two-week intervals. Serum samples were collected via retro-orbital puncture prior to the initial immunization (establishing a true seronegative baseline) and two weeks following the final booster. Extracted sera were immediately clarified by centrifugation and stored at −80 C to prevent antibody degradation.

### 2.7 *In Vitro* infectivity assay

HEK293TT cells were accurately seeded at a density of 3 × 10^4^ cells per well in 96-well flat-bottom microplates (SPL Life Sciences). To establish reliable baseline infectivity across the panel, PsVs were applied at a standard 1:100 dilution. Simultaneously, dose-response linearity and analytical thresholds were rigorously evaluated through 10-fold serial dilutions. At precisely 72 hours post-infection, cLuc activity was quantified directly from the supernatant using the Pierce™ *Cypridina* Luciferase Flash Assay Kit (Thermo Fisher Scientific), while conventional SEAP expression was measured via the Great EscAPe™ SEAP Chemiluminescence Kit 2.0 (Takara Bio) following heat treatment.

### 2.8 *In Vitro* pseudovirus-based neutralization assay

PBNAs were performed with optimized modifications to enhance throughput and reliability. Serially diluted hyperimmune serum samples (four-fold increments) were pre-incubated with predetermined, normalized infectious doses of the 28 cLuc- or SEAP-HPV PsVs. To fully facilitate stable antibody-virus complex formation, these mixtures were continuously agitated at 4 C for 1 hour on a rocking shaker. Subsequently, the neutralized complexes were transferred onto pre-seeded HEK293TT target cells and incubated for 72 hours at 37 C. Neutralizing antibody titers were determined by quantifying the residual viral reporter activity, employing the cLuc and SEAP detection methodologies detailed above.

### 2.9 *In Vivo* murine cervicovaginal challenge model and bioluminescence imaging

Because the intact, keratinized murine vaginal epithelium is naturally highly resistant to HPV penetration, mice were subcutaneously administered 3 mg of medroxyprogesterone acetate (Depo-Provera; Pfizer) three days prior to viral challenge (14). This potent hormonal synchronization arrests the estrous cycle in a diestrus-like phase, significantly thinning the mucosal epithelial layer and rendering the basement membrane susceptible to viral binding. On the day of infection, the vaginal mucosa was lightly mechanically abraded using a cytobrush to simulate human microtrauma, followed immediately by intravaginal inoculation with 20 µL of cLuc-HPV PsVs suspended in a viscous 3% carboxymethyl cellulose (CMC) formulation to prevent viral leakage. Three days post-infection, 20 µL of vargulin substrate (10 µg/animal; NanoLight Technology) was gently introduced into the vaginal cavity. High-resolution bioluminescent signals were captured continuously over a 10-minute integration period using the advanced Xenogen IVIS 100 imaging system (Xenogen) and spatially quantified via Living Image 2.5 software (PerkinElmer). The strict background threshold for positive mucosal infection was defined as the mean average radiance of mock-infected controls plus two standard deviations (Mean + 2 SD).

### 2.10 Statistical analysis

All complex statistical evaluations and non-linear curve fittings were performed using GraphPad Prism version 11 (GraphPad Software). Data are robustly presented as the mean ± standard error of the mean (SEM) of multiple independent replicates. Statistical significance between groups was assessed utilizing either an unpaired Student’s t-test or one-way analysis of variance (ANOVA) followed by Tukey’s rigorous *post hoc* test for multiple comparisons. IC50 metrics were mathematically extrapolated using standardized non-linear regression analysis. Inter-assay consistency and operational reliability were expressed as the coefficient of variation (%CV). Methodological concordance between the cLuc and SEAP platforms was determined via Pearson’s correlation coefficient (r) and the coefficient of determination (*R*²). An alpha level of *p*<0.05 was prospectively defined as denoting statistical significance.

## 3 Results

### 3.1 Strategic design and operational optimization of the cLuc-HPV platform

To effectively address the throughput constraints and analytical bottlenecks inherent in conventional serological assays, we engineered a high-fidelity PsV library deploying cLuc as a multifaceted, dual-function reporter. The architectural design of this platform explicitly targeted three major scientific imperatives: (i) the complete elimination of the cumbersome and technically variable 65 C heat-inactivation step, (ii) the extensive expansion of the analytical scope to encompass 28 distinct oncogenic and low-risk HPV genotypes critical for multivalent vaccine assessment, and (iii) the assurance of dual-utility, providing high biochemical sensitivity for *in vitro* assays alongside robust spatial resolution for *in vivo* monitoring.

**Figure 1:**
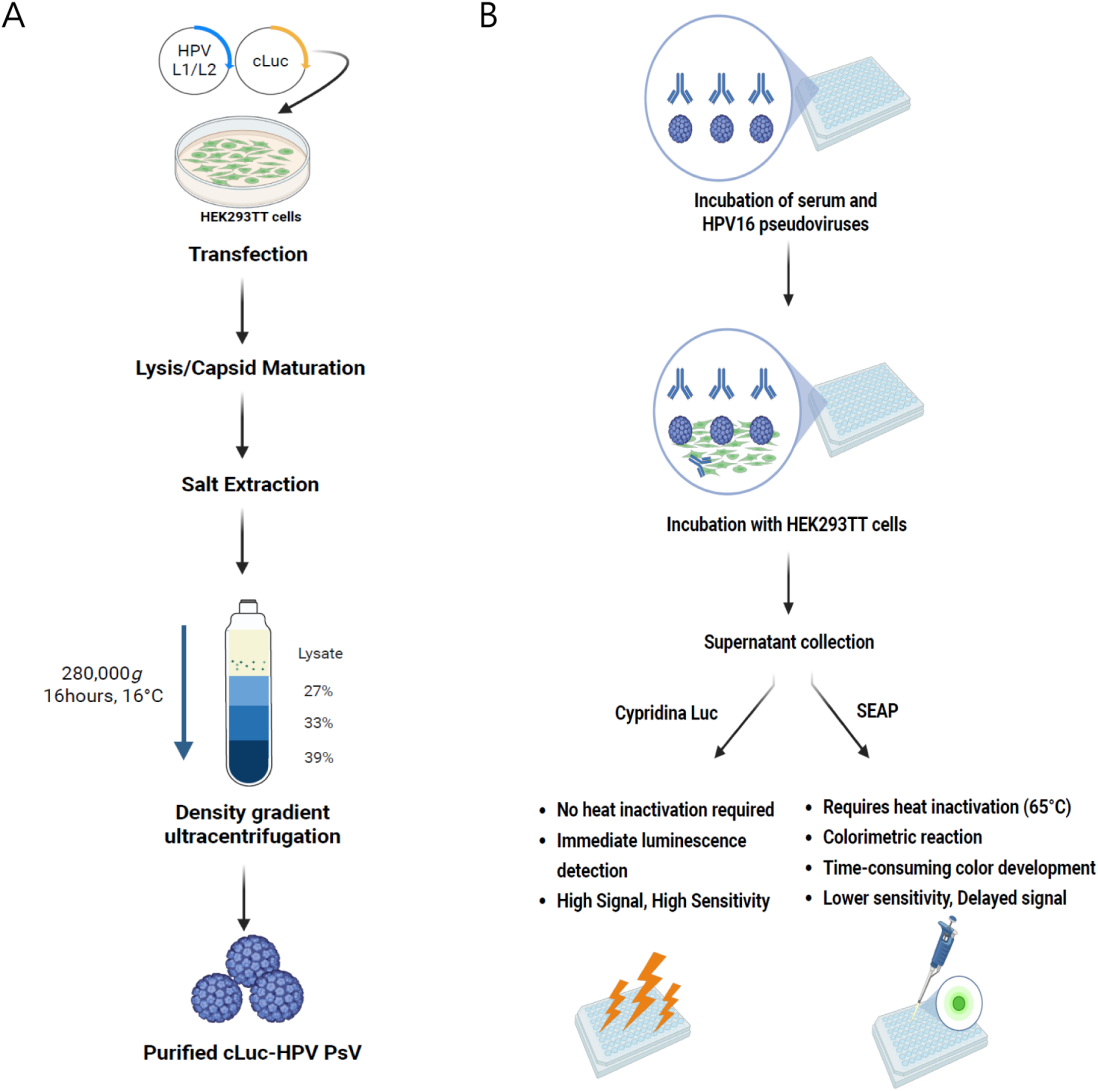
Schematic overview of the cLuc-HPV pseudovirus production and the comparative neutralization assay workflow. **(A)** Generation and purification of cLuc-encapsidating HPV PsVs. HEK293TT cells are co-transfected with plasmids encoding HPV L1 and L2 capsid proteins alongside the cLuc reporter plasmid. Following cell lysis, maturation, and salt extraction, mature PsVs are purified using a three-step density gradient ultracentrifugation. **(B)** Workflow of the PBNA and a methodological comparison between cLuc and SEAP reporter systems. Serum samples and PsVs are pre-incubated to facilitate virus-antibody binding prior to the infection of HEK293TT cells. After incubation, culture supernatants are harvested for reporter detection. The cLuc platform offers procedural advantages over the SEAP assay, including the omission of the heat inactivation step, immediate measurement, and enhanced signal sensitivity.

The standardized generation workflow (**Figure 1A**) encompassing precise plasmid co-transfection, a 24-hour detergent-mediated *in vitro* maturation phase, and high-resolution density gradient ultracentrifugation consistently yielded highly pure, structurally intact virions across the entire 28-genotype panel. The selection of cLuc was predicated on its uniquely advantageous biochemical partitioning: ATP-independent extracellular secretion (70–80%) combined with a fraction of stable intracellular retention (20–30%). Biologically, this dual profile permits rapid, non-destructive supernatant sampling for high-throughput *in vitro* screening, while simultaneously reserving sufficient intracellular luminescent capacity to enable reliable bioluminescent tracking of infected mucosal tissues in living animal models.

The operational efficiency of this advanced methodology is most clearly highlighted by the contrast in processing time compared to the traditional SEAP assay (**Figure 1B**). SEAP protocols require a 30-minute thermal inactivation at 65 C to quench heat-labile endogenous mammalian phosphatases, followed by an hour-long colorimetric development. This results in a 100–120 minute processing window per multi-well plate. Conversely, because cLuc originates from a marine ostracod and lacks any endogenous mammalian orthologs, it inherently escapes background biological interference. This lack of background enables instantaneous, direct luminescence detection, significantly reducing the readout phase to under 10 minutes per plate. By eliminating thermal-induced experimental artifacts (such as edge effects during heating) and minimizing workflow duration, the cLuc platform provides the processing capacity essential for the high-throughput preclinical screening of large clinical trial cohorts.

### 3.2 Comprehensive assembly and structural fidelity of the 28-Type PsV library

Validating the structural fidelity and correct biomolecular assembly across a widely divergent phylogenetic spectrum is essential to guarantee the reliability of any newly developed neutralization system. To address this, we expanded our cLuc reporter platform to encapsulate an extensive library of 28 distinct HPV PsVs, capturing a wide array of epidemiologically significant mucosal and cutaneous strains that represent the next frontier in vaccine development.

**Figure 2:**
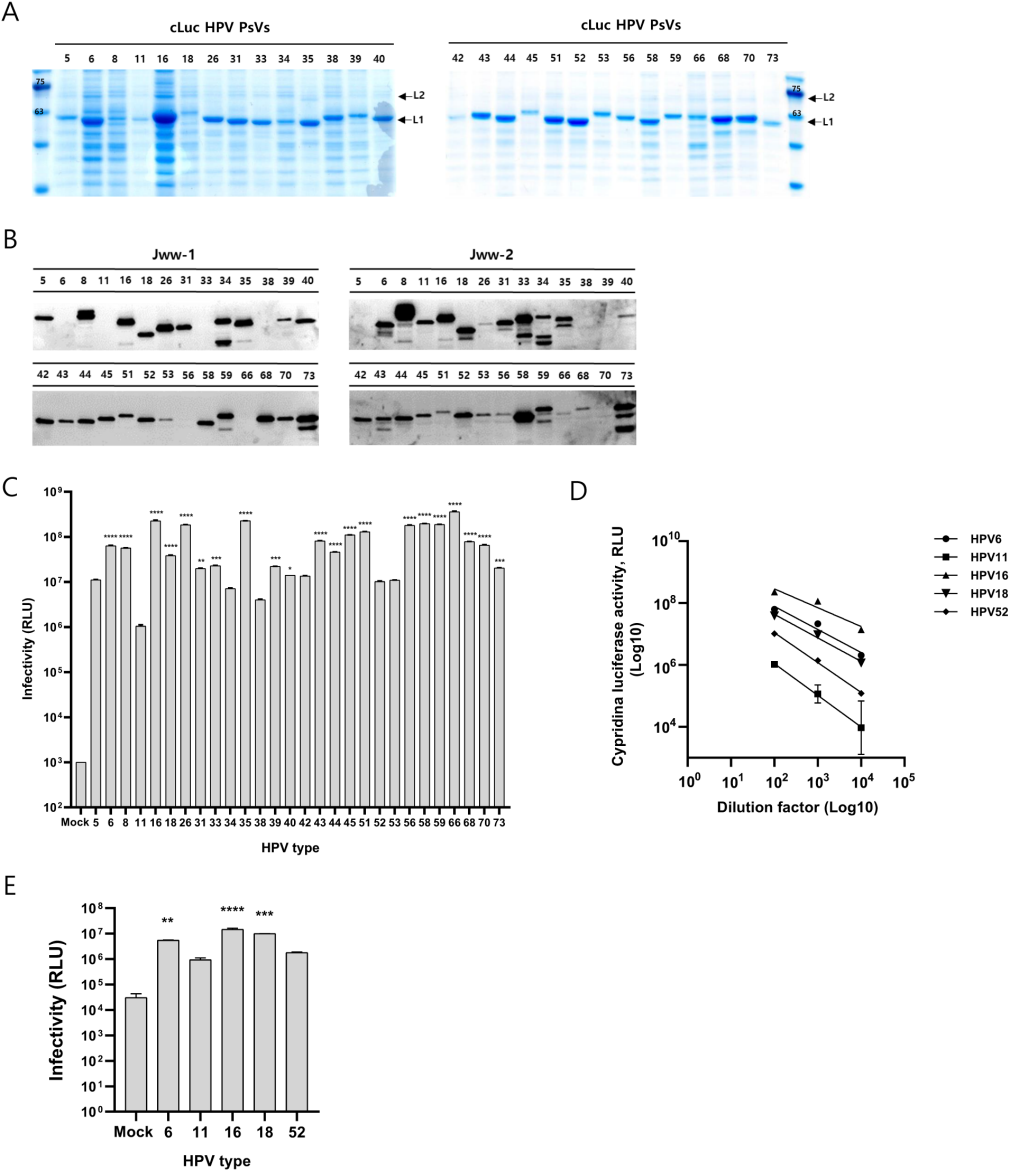
Production, structural characterization, and infectivity validation of the 28 distinct cLuc-HPV pseudoviruses (PsVs). **(A)** SDS-PAGE analysis of the purified cLuc-HPV PsVs. The gels were stained with Coomassie brilliant blue to visualize the total protein composition. Black arrows indicate the assembly and incorporation of the major (L1, ~55 kDa) and minor (L2, ~72 kDa) viral capsid proteins across all 28 HPV genotypes. **(B)** Western blot analysis confirming the presence of the L2 capsid protein. The purified PsVs were immunoblotted using the laboratory-generated monoclonal antibodies, JWW-1 (left panels) and JWW-2 (right panels), which target the HPV L2 region. **(C)** *In vitro* infectivity screening of the 28 cLuc-HPV PsVs. HEK293TT cells were infected with the PsVs at a 1:100 dilution, and the cLuc activity (Relative Light Units, RLU) was measured at 72h post-infection. **(D)** Dose-dependent *in vitro* infectivity of five representative high- and low-risk HPV genotypes (HPV 6, 11, 16, 18, and 52). Serial dilutions of the PsVs demonstrated a linear correlation between the viral dilution factor and the resulting luminescence signal. **(E)** *In vitro* infectivity validation of the SEAP-HPV PsVs. HEK293TT cells were infected with five representative SEAP-HPV PsV genotypes (HPV 6, 11, 16, 18, and 52) at a 1:100 dilution, and the SEAP chemiluminescence signals (RLU) were measured at 72h post-infection. Quantitative data are expressed as the mean ± SEM of independent experiments. Statistical significance compared to the mock-infected control was determined using an unpaired, two-tailed Student’s t-test (*p<0.05, **p<0.01, ***p<0.001, ****p <0.0001).

SDS-PAGE profiling (**Figure 2A**) confirmed the successful and consistent encapsidation of the essential viral architecture across all 28 genotypes. The presence of the L1 major capsid protein (~55 kDa), which auto-assembles into the 72 pentameric capsomeres forming the icosahedral backbone, was evident. Similarly, the stoichiometrically appropriate bands of the L2 minor capsid protein (~72 kDa) were present in all samples. Because nature dictates a ratio of exactly 360 L1 molecules to a maximum of 72 L2 molecules per virion, the relative band intensities confirmed that our synthetic generation pipeline reflects native biological virion assembly (15).

To verify the correct spatial incorporation of L2 into the functional capsids, immunoblotting was performed utilizing the custom JWW-1 and JWW-2 monoclonal antibodies. These engineered immunoglobulins are specifically designed to target a conserved N-terminal epitope of the L2 protein, a domain essential for viral endosomal escape during natural infection. Despite their initial developmental origins targeting the HPV16 sequence, both antibodies exhibited broad cross-reactivity with the tire28-type library. Notably, HPV38 was the singular exception, showing no detectable signal in the immunoblot analysis (**Figure 2B**). This broad immunoreactivity provides strong molecular evidence that our synthesized cLuc-PsVs possess the critical structural motifs requisite for driving receptor-mediated cellular entry mechanisms.

### 3.3 Robust *In vitro* infectivity and expansive analytical linearity

Following structural validation, the baseline infectivity of the 28-type library was evaluated utilizing HEK293TT cells. These target cells are permissive to HPV pseudovirus entry due to their dense surface expression of heparan sulfate proteoglycans (HSPGs), the primary attachment receptors for HPV. Relative to mock-infected biological controls, each genotype in the 28-type panel successfully attached, internalized, and established active gene expression, generating luminescent signals that substantially exceeded baseline background thresholds by at least three orders of magnitude. The most infectious strains surpassed the background by over five orders of magnitude (**Figure 2C**). Specifically, at a standardized 1:100 dilution, emitted signals ranged from 10^6^ to 4 × 10^8^ relative light units (RLU). Although natural biological variances in packaging efficiency, capsid thermodynamic stability, and L2-mediated entry kinetics resulted in inter-genotypic signal fluctuations, every tested virus exceeded the analytical detection limit required for downstream neutralization studies.

To evaluate the analytical sensitivity and quantitative precision of the assay, a serial dilution matrix (10², 10³, and 10^4^) was systematically applied to five representative genotypes (HPV 6, 11, 16, 18, and 52). The resulting flash-luminescence displayed dose-dependent linearity, yielding significant correlation coefficients (*R*²) ranging from 0.83 to 0.99, alongside non-zero slopes (*P*<0.05) across all types (**Figure 2D**). This linear dynamic range is critical; it ensures that the cLuc biochemical system tireresists optical saturation at peak viral loads while maintaining precise signal resolution at low viral concentrations. This linearity is a prerequisite for calculating accurate, reproducible IC_50_ metrics in concentrated hyperimmune sera. Parallel comparative validation utilizing conventional SEAP-PsVs confirmed satisfactory baseline infectivity at the same dilutions, establishing a comparative framework for subsequent direct titrations (**Figure 2E**).

### 3.4 Enhanced analytical sensitivity and superior reproducibility of the cLuc PBNA

**Figure 3:**
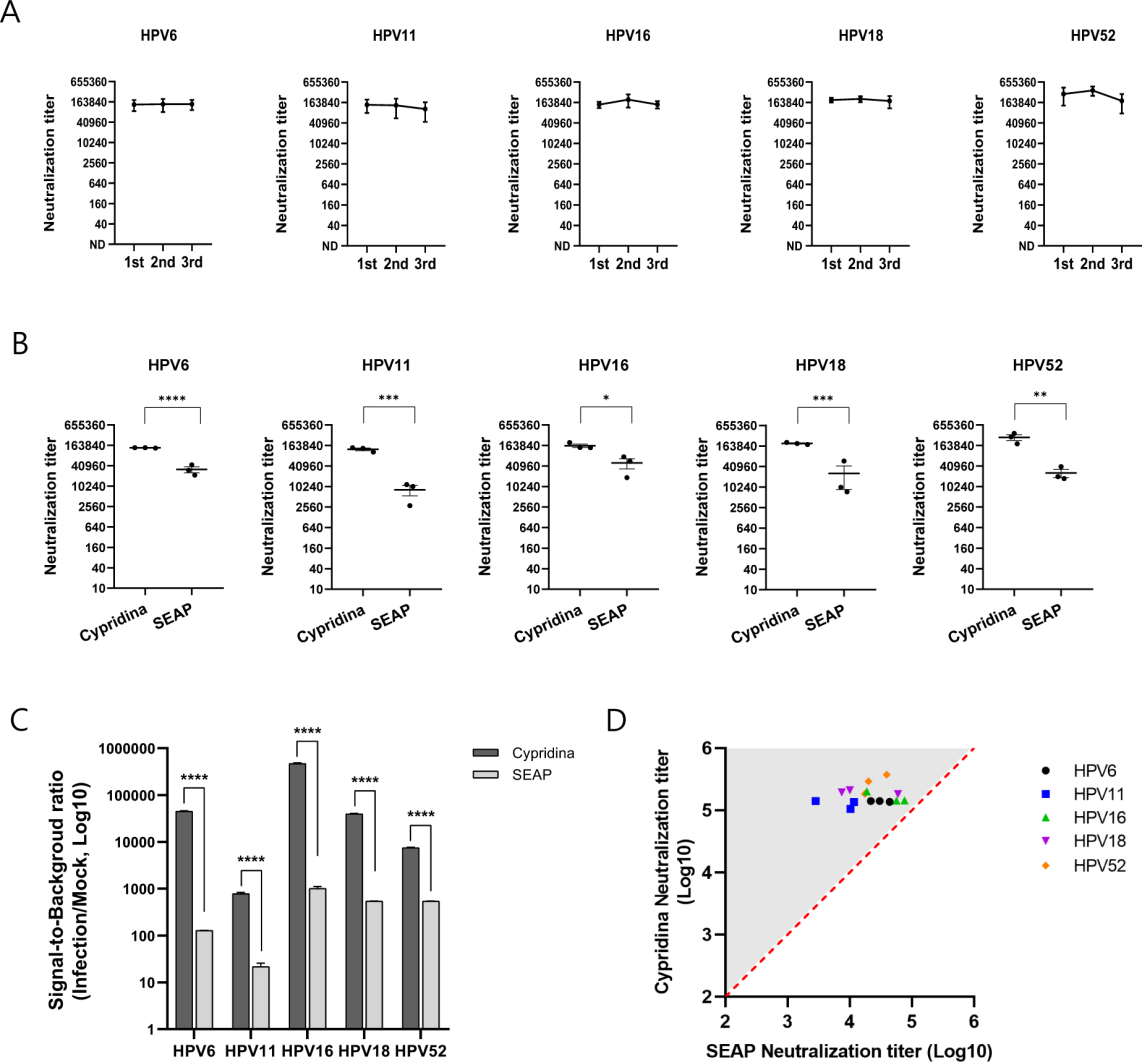
Validation and comparative analysis of the novel cLuc-based HPV pseudovirus neutralization assay. **(A)** Inter-assay reproducibility of the cLuc-based neutralization assay. The IC_50_ neutralizing titers against five representative HPV genotypes (HPV 6, 11, 16, 18, and 52) were evaluated across three independent experimental runs. Individual data points represent IC_50_ values from each run, demonstrating consistent performance (detailed %CV in **Table 1**). **(B)** Head-to-head comparison of IC_50_ neutralizing titers determined by the cLuc and the conventional SEAP platforms. Data are expressed as the mean ± SEM of three independent experiments. Statistical significance was determined using an unpaired, two-tailed Student’s t-test (*p < 0.05, **p < 0.01, ***p < 0.001, ****p < 0.0001). **(C)** Comparison of signal-to-background ratios between the cLuc and SEAP systems. The SBR was calculated as the ratio of the luminescence signal from infected cells to that of mock-infected controls. Statistical significance was determined using a two-way ANOVA followed by Sidak’s multiple comparisons test on Log_10_-transformed data (****p < 0.0001). The cLuc system provides a significantly higher SBR across all genotypes, facilitating more precise titer determination. **(D)** Correlation analysis of Log_10_-transformed neutralization titers between the cLuc and SEAP assays. Individual HPV genotypes are distinguished by specific shapes and colors. The red dashed line represents the line of identity (y = x). Data points are positioned above the identity line, indicating the enhanced sensitivity of the cLuc-based assay. The strength of the association was evaluated using Pearson’s correlation coefficient (*R*² > 0.99, *p* < 0.0001).

To facilitate a statistically powered comparative evaluation between the cLuc and traditional SEAP platforms, hyperimmune sera were generated by systematically immunizing BALB/c mice with adjuvanted, multivalent VLPs. Initially, we sought to assess the inter-assay reproducibility of the novel cLuc system. As detailed in **Table 1** and **Figure 3A**, the cLuc platform delivered consistent, grouped IC_50_ neutralizing titers across three independent experimental iterations performed on different days. The calculated coefficients of variation (%CV) ranged from 1.74% to 33.32%. Crucially, the maximum fluctuation observed in HPV 52 (33.32%) remained within the 3-fold biological variance that is accepted for cell-based serological and virological assays. This constrained variance affirms the platform’s operational stability and suitability for validated screening.

**Table 1:** Inter-assay reproducibility of the cLuc-based HPV pseudovirus neutralization assay.

HPV genotype	Mean IC50 titer	Standard deviation (SD)	Inter-assay %CV
HPV 6	139,003	2,415	1.74%
HPV 11	127,035	19,128	15.06%
HPV 16	162,050	32,775	20.23%
HPV 18	195,570	14,081	7.20%
HPV 52	283,226	94,358	33.32%

In a direct analytical comparison, the cLuc assay reproducibly detected higher neutralizing titers than its SEAP counterpart across all evaluated genotypes (*P*<0.05, **Figure 3B**). This enhanced detection capacity is directly attributable to the signal-to-background ratio (SBR) generated by the cLuc reporter. The cLuc SBR significantly exceeded that of SEAP by up to three orders of magnitude (*P*<0.0001, **Figure 3C**). In the context of IC_50_ calculations, this enhanced SBR improves the precision of mathematical non-linear curve fitting, particularly at the lower asymptotes of serum dilutions where weak SEAP signals typically decay into endogenous background noise.

Furthermore, linear regression analysis performed on the Log_10_-transformed neutralization titers corroborated a significant functional concordance between the two distinct methodologies (*R²* > 0.99, *P*<0.0001, **Figure 3D**). Most importantly, the spatial positioning of all analyzed data points above the 1:1 identity line clearly demonstrates the marked superior analytical sensitivity of the cLuc system in detecting low-abundance neutralizing antibodies, a feature critical for evaluating long-term vaccine waning.

### 3.5 Translational *In vivo* validation via murine cervicovaginal challenge

**Figure 4:**
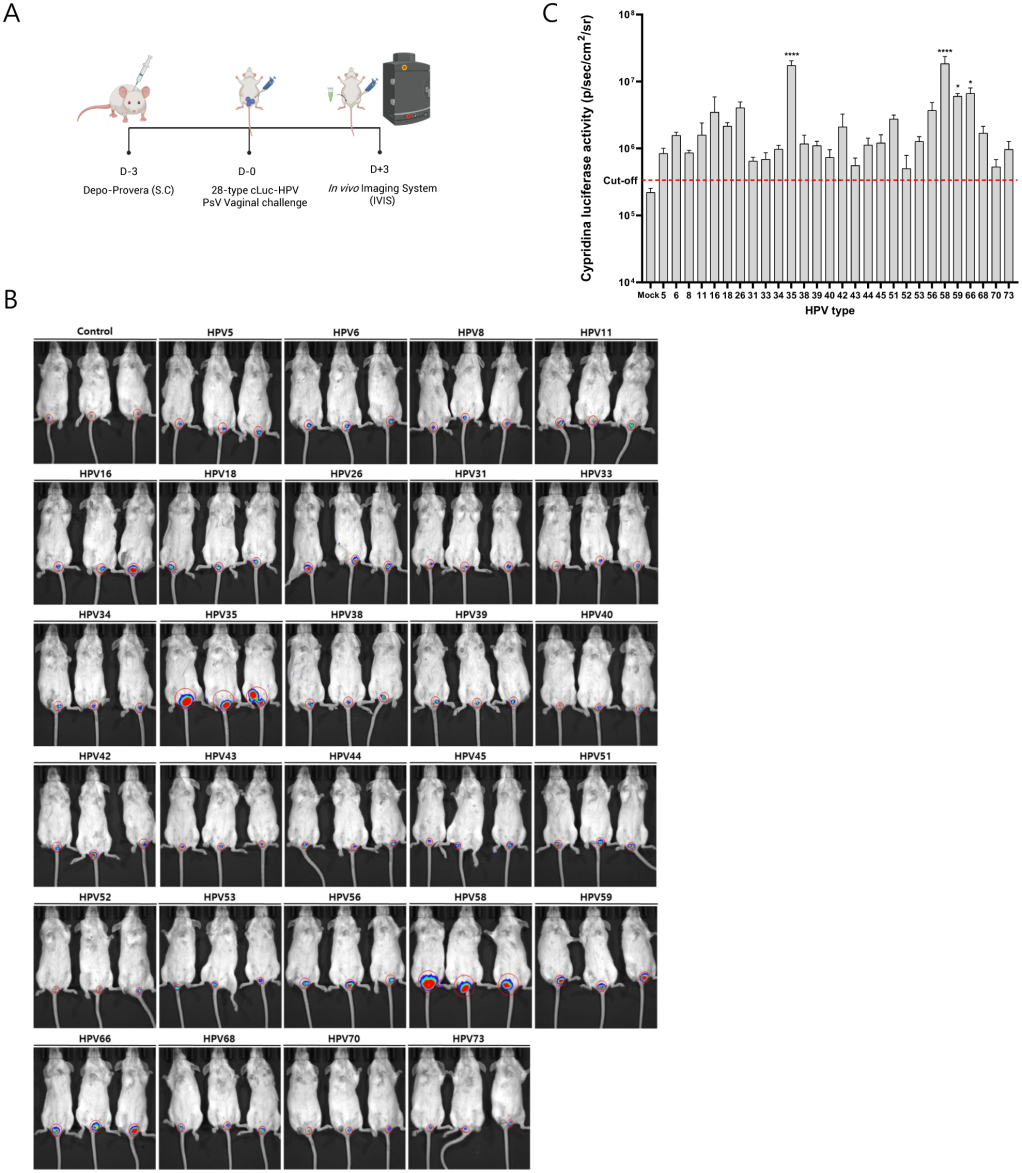
*In vivo* infectivity screening of the 28-type cLuc-HPV pseudovirus (PsV) panel using a murine cervicovaginal challenge model. **(A)** Schematic representation of the *in vivo* experimental timeline. Female BALB/c mice were pre-treated with medroxyprogesterone acetate (Depo-Provera; S.C.) on Day -3 to synchronize the estrous cycle and increase mucosal susceptibility. On Day 0, the vaginal mucosa was mechanically abraded, followed by challenge with the 28 cLuc-HPV PsVs. *In vivo* bioluminescence imaging was performed on Day 3 post-infection. **(B)** Representative *in vivo* bioluminescence images of the murine cervicovaginal tracts at 3 days post-infection. The pseudo-color heat maps indicate the spatial distribution and relative intensity of the cLuc signals emitted from the infected mucosal tissues. **(C)** Quantitative analysis of the in vivo viral infectivity. The bioluminescence signal intensity is expressed as average radiance (photons/sec/cm^2^/sr). The red dashed line indicates the background threshold, calculated as the mean + 2 SD of the mock-infected control. Quantitative data are presented as the mean ± SEM of independent animal subjects. Statistical significance compared to the mock-infected control was evaluated using an unpaired, two-tailed Student's t-test (*p<0.05, ****p<0.0001).

To validate the preclinical translatability and utility of the cLuc platform for *in vivo* biological applications, we deployed a Depo-Provera-sensitized murine cervicovaginal challenge model (**Figure 4A**). Because the keratinized murine vaginal tract is naturally resistant to HPV, hormonal synchronization with Depo-Provera induces a diestrus-like thinning of the mucosal epithelium. Following cytobrush-mediated mechanical abrasion to simulate sexual microtrauma and expose the basement membrane, the mice were intravaginally inoculated with formulations of each of the 28 cLuc-HPV PsVs.

At 3 days post-infection, non-invasive bioluminescence imaging confirmed viral entry, uncoating, and reporter expression localized to the lower cervicovaginal reproductive tract. In contrast, mock-infected negative controls displayed minimal background activity (**Figure 4B**). Quantitative spatial analysis (**Figure 4C**) revealed that all 28 genotypes penetrated the tissue and established quantifiable *in vivo* infections, with emitted radiances significantly surpassing baseline background thresholds. This multi-genotype *in vivo* dataset confirms the biochemical sensitivity of the cLuc reporter in living mammalian tissues and validates that its 20–30% intracellular retention fraction provides the requisite spatial resolution for localized *in vivo* tracking. This establishes the platform’s utility for evaluating the *in vivo* prophylactic efficacy of mucosal vaccines in animal models.

## 4 Discussion

In response to the growing global demand for high-throughput and precise evaluation of emerging multivalent HPV vaccines, we developed, biochemically optimized, and validated a versatile cLuc-based PsV platform. Encompassing a comprehensive library of 28 distinct mucosal and cutaneous HPV genotypes, this biological system effectively bridges the critical gap between high-throughput *in vitro* serological screening and non-invasive *in vivo* spatiotemporal tracking.

For nearly two decades, the SEAP-based PBNA has served as the serological gold standard for neutralizing antibody assessment. However, its strict reliance on a 65 C thermal inactivation step to mitigate endogenous mammalian alkaline phosphatases introduces critical workflow bottlenecks. Frequently, residual phosphatase activity survives this heat treatment, generating persistent and variable background interference that degrades analytical precision, particularly when evaluating dose-sparing vaccine schedules that may elicit lower initial antibody titers. Our strategic incorporation of the cLuc reporter circumvents this fundamental vulnerability. As an exclusively non-mammalian enzyme derived from marine ostracods, cLuc exhibits negligible background activity in both murine and human biological matrices. This purity enables instantaneous, modification-free luminescent readings directly from raw samples. Consequently, our analytical data demonstrate a signal-to-background ratio (SBR) that is up to five orders of magnitude superior to traditional methods (**Figure 3**). This improvement in signal clarity provides the mechanistic basis for the cLuc PBNA to detect higher and more accurate neutralizing antibody titers compared to the legacy SEAP standard.

A primary strength of our platform is its expansive linear dynamic range, which ensures the unbiased and mathematically sound quantification of neutralizing efficacy across a diverse array of genotypes. It is well documented that natural biological variations in viral packaging efficiency, thermodynamic stability, or HSPG receptor-binding kinetics result in disparate baseline luminescence intensities among the 28 types. However, these inherent virological differences do not artificially skew the resulting neutralization readouts. By anchoring the functional analytical readout to the linear phase of the viral dose-response curve (**Figure 2D**), our platform ensures that vaccine-induced immunogenicity can be compared impartially and accurately across a heterogeneous virological spectrum. The structural consistency of this comprehensive panel was verified via the cross-reactive JWW-1 and JWW-2 antibodies, confirming the stoichiometric preservation and functional presentation of the L2 minor capsid protein across all 28 distinct strains (11).

Furthermore, the translational power of this platform was realized in the murine cervicovaginal challenge model (**Figure 4**). While secreted reporters like SEAP diffuse rapidly from mucosal infection sites, blurring spatial boundaries and anatomical resolution, the naturally occurring 20–30% intracellular retention of cLuc anchors the luminescent signal to the primary anatomical foci of viral entry. Although this fractional retention was sufficient to statistically and visually differentiate infected mucosal cohorts from mock controls, future targeted biological engineering aimed at modifying the cLuc signal peptide to shift the pharmacokinetic ratio closer to a 50:50 (secreted:retained) equilibrium could potentially enhance deep-tissue signal resolution without sacrificing the requisite *in vitro* supernatant sensitivity.

While the comprehensive biological study presented herein establishes a robust and validated framework, we acknowledge that certain technical aspects warrant further refinement. Specific divergent genotypes, such as HPV 43 and 70, naturally exhibited comparatively lower luminescent yields than oncogenic strains like HPV 16 or 18. Although these emitted signals remained quantifiable and statistically valid, optimizing the reporter plasmid architecture—perhaps via the insertion of alternative promoter systems or specialized mRNA stabilization motifs—could boost and normalize signal strength uniformly across the entire 28-type panel. Furthermore, expanding this validated framework to evaluate longitudinal clinical trial cohorts utilizing human sera is the critical next step (16). Implementing this sensitive platform in clinical settings will be essential in precisely defining the serological correlates of protection against emergent oncogenic variants that may arise due to vaccine-induced evolutionary pressure.

## 5 Conclusion

In summary, this study establishes a sensitive, operationally streamlined, and validated cLuc-based HPV pseudovirus platform comprising 28 distinct clinical genotypes. By systematically eliminating the thermal inactivation bottlenecks and biological interference that affect conventional assays, this advanced system provides a high-throughput capability suited for large-scale serological profiling and epidemiological surveillance. Driven by an improved, artifact-free signal-to-background ratio, the cLuc PBNA offers enhanced analytical sensitivity, enabling a more precise and comprehensive evaluation of neutralizing antibody kinetics. Furthermore, the successful implementation of this dual-function platform in a non-invasive mucosal challenge model expands its preclinical utility. Consequently, this versatile and scalable technology offers an essential analytical framework to accelerate the robust evaluation and global deployment of next-generation multivalent HPV vaccines and targeted therapeutic interventions.

## Data Availability

The original contributions presented in the study are included in the article/supplementary material. Further inquiries can be directed to the corresponding author.
